# Performance profiles of professional female tennis players in grand slams

**DOI:** 10.1371/journal.pone.0200591

**Published:** 2018-07-19

**Authors:** Yixiong Cui, Miguel-Ángel Gómez, Bruno Gonçalves, Jaime Sampaio

**Affiliations:** 1 Faculty of Physical Activity and Sport Sciences–INEF, Technical University of Madrid, Madrid, Spain; 2 Research Center in Sports Sciences, Health Sciences and Human Development (CIDESD), CreativeLab Research Community, Universidade de Trás-os-Montes e Alto Douro, Vila Real, Portugal; Universita degli Studi di Verona, ITALY

## Abstract

**Introduction:**

The aim of the study was to (i) analyze the match performance of professional female tennis players in different Grand Slams; (ii) model the relationships between match performance variables and relative quality; and (iii) build typical performance profiles for those players in Grand Slams.

**Method:**

Data of a total of 1369 matches were collected within 2014–2017 four Grand Slams (Australian Open: n = 499; Roland Garros: n = 249; Wimbledon: n = 249 and US Open, n = 372). Correlations between 37 performance variables and relative quality (difference of expected rounds between two competing players of given ranking) were determined and automatically classified into two groups of magnitudes via two-step cluster analysis. Higher-correlated variables were used to build players’ typical performance profiles via regression-based technique to give percentage evaluation scores (%ES), which means the percentage of matches where a performance variable value would be expected to be lower than the observed value considering the RQ of two competing players.

**Results:**

Players had more service winners, double faults, return winners and return unforced errors in the Australian Open and US Open, implying a “fast-fast” serve strategy, and higher dominance ratio and better serving performance in Wimbledon. While receiving players had better chances to break opponents’ service game in Roland Garros. Distance covered became similar in all Grand Slams. All studied variables showed obvious correlation with RQ expect for those of physical performance.

**Conclusions:**

The findings (i) indicate that female game in Grand Slams remained to be a contest over baseline, although players had good efficiency at net; (ii) demonstrate the influence of relative quality on serve and return, break point, net point and efficiency performance; and (iii) evidence the usefulness of applying %ES to evaluate performance of individual player.

## Introduction

The competiveness of four tennis Grand Slams (Australian Open, Roland Garros, Wimbledon and US Open) that are held every year represents the maximum level of competition of this sport and its progression [1, 2]. Each tournament requires the player to play with a draw of 128 competitors within a period of two weeks if they aspire to win the final trophy, higher ranking points or prizes [3]. Therefore, the importance of those slams raised a great number of studies on how players performed in those events and provided valuable feedback for coaching and training in tennis [4–11].

The difference of court surfaces in four Grand Slams determines that players adjust their strategies on a slam basis [8] and it is necessary to understand how they respond accordingly in different events. Few studies have made thoroughly an examination of how professional female players performed in four Grand Slam events [8, 12], not to mention how they performed in different court surfaces [13]. O’Donoghue and Ingram (2001) reported the match time and rally patters in four Grand Slams by using notational data and O’Donoghue (2002) analyzed the game in the Australian Open based on match outcome. Recently Reid et al (2016) described the match-play characteristics in the Australian Open with data from Hawk-eye tracking system. Many of those studies just considered the performance of female players in certain Grand Slam like Australian Open [14] and failed to compare that with other slams.

In general, most current studies are focused on the performance of male players from a physiological perspective. It was reported that players experienced a decrease in body mass and blood lactate while an increase in heart rate, perception of effort and salivary cortisol concentrations on a set-to-set basis during the match, which showed the increasing fatigue and stress male players had to cope with under real match scenario [15, 16]. Meanwhile, the movement patterns [17, 18] and the technical and tactical effectiveness of male players were also widely investigated [6, 13, 19, 20]. The previous studies found that first and second serve capability (direction, speed and success), breakpoint opportunity, net point success and distance covered within the match were important predictors to their success and the differentiation of player types. Consequently, there is a need to update the understanding of female players’ performance in Grand Slams, particularly by identifying their current profiles with recommended approaches [21, 22].

The existing normative performance profiling technique [23] proposed to describe tennis performance uses the median, lower and upper quartiles of normalized performance indicators to represent the performance of a group of players or single player, and maps them onto a radar chart. This not only facilitate to an instant visualization of player’s performance profile across various performance indicators which were originally in different units, but helps to inspect or compare the consistency and inconsistency in performance between determined players against certain percentile banding of population of interest [22, 24]. However, criticism of this approach is that there is a risk of misinterpreting individual performance if the quality of opponent is not included in the player profiles [21]. In response to the limitations, O'Donoghue and Cullinane (24) brought out a new profiling technique that can properly evaluate and interpret the performance of individual tennis players while taking into account rivals of different strength. The approach considers primarily the relative quality (RQ) of two tennis players that compete in a match, utilizing their 52-week world ranking, and then it provides percentage evaluation scores (%ES) of each performance variable to interpret how good or bad players performed when they are against different opponents. The usefulness of the technique was evidenced in profiling individual performance, typical performance and performance trends in tennis. However, in the study of O'Donoghue and Cullinane (24), there were few performance variables used to establish the profile of professional male tennis players in the Australian Open. The correlations between other performance variables and relative quality remain unknown not only for male players, but also for female players within four Grand Slams, considering the effects of different courts.

As match-related statistics can provide insightful information on players’ perception and action. Former studies tended to analyze the performance variables in isolation: assessing solely serve and return performance variables likes aces, first serve points in, first serve points won, second serve points won [25]; or break point performance variables [6]; or physical performance variables [26]. In order to address this issue, it is necessary to bring together all performance variables of interest and to evaluate them comprehensively if we want to represent the performance of elite female players in different slams [22].

Therefore, the aim of the present study was to: (i) analyze and compare the match performance of professional female tennis players in recent four Grand Slams; (ii) model the relationships between match performance variables and relative quality; and (iii) build typical performance profiles for female players in Grand Slams.

## Method

### Sample and data source

Data from 1369 matches played in four Grand Slams women’s singles were obtained from separate official websites, which consisted of 499 matches from Australian Open (2014–2017, www.ausopen.com), 249 matches from Roland Garros (2015–2016, www.rolandgarros.com), 249 matches from Wimbledon (2015–2016, www.wimbledon.com) and 372 matches from US Open (2014–2016, www.usopen.org). Walkover matches or those with players who retired were excluded from the study. The data included the match statistics from 257 unique players (2738 observations) and their 52-week world rankings (ranking range: 1 to 992) in the corresponding slam. As the names of some players are identified in different formats across different Grand Slams, all the players observations were checked after the data collection and their names and surnames were unified among all events in order to match the unique player observations. The study was approved by the research commission of the Faculty of Physical Activity and Sports Sciences (Technical University of Madrid) and all procedures are conducted following the European Data Protection Law in order to maintain the anonymity of sampled players.

### Variables and operational definitions

**Fig 1:**
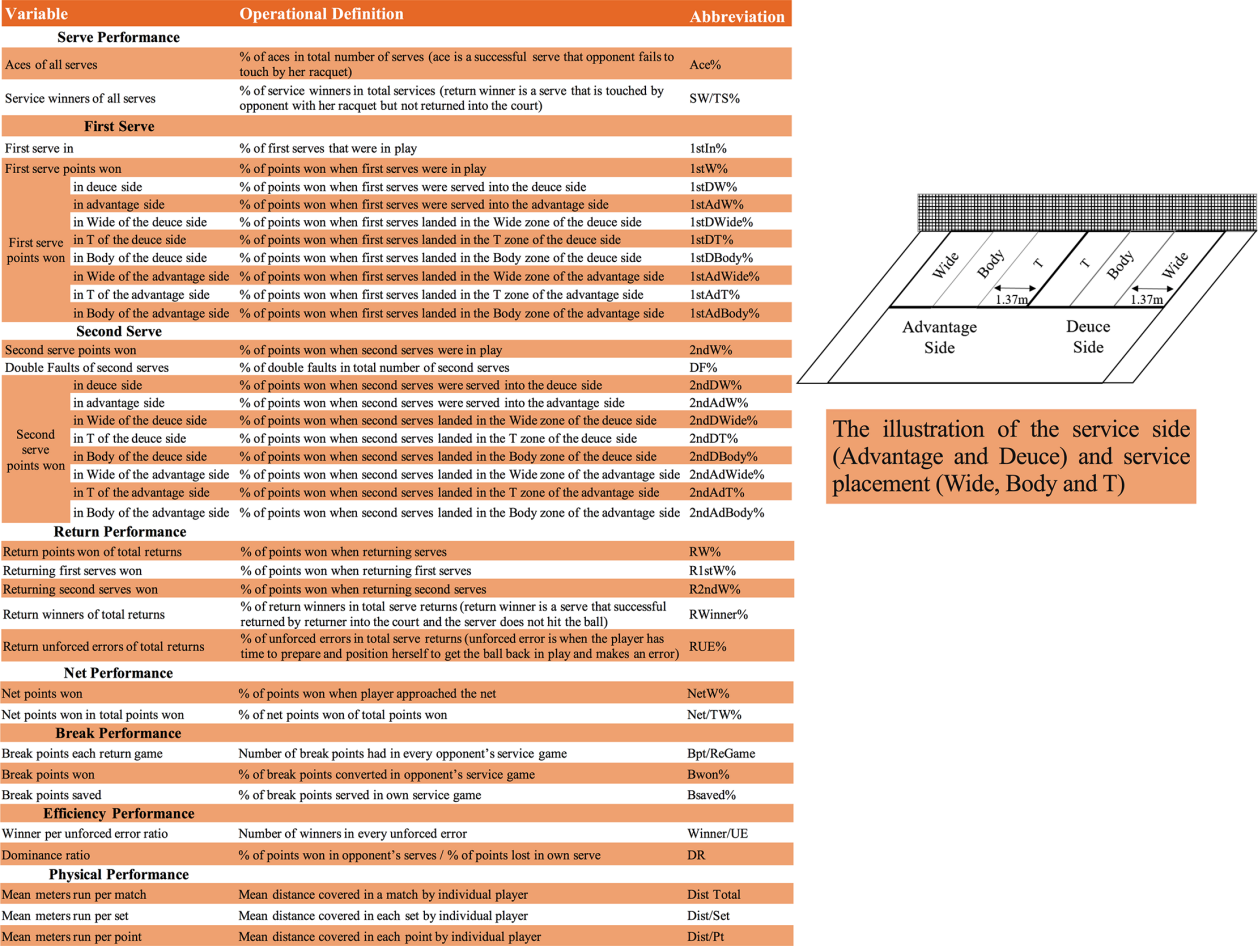
Match performance variables, operational definitions and abbreviations.

The study included 37 variables that were selected from the raw data and Fig 1 shows their names and operational definitions.

It was impossible to assess the reliability of the tracking data (i.e. mean meters run per match/set/point) by Hawk-eye system, although being used in previous studies [9, 10]. Therefore, two experienced performance analysts in tennis observed and collected the non-tracking data (all notational variables shown in Fig 1 excluding physical performance-related variables) of two matches that were randomly selected. Afterwards, comparisons were made between inter-observers over all variables. The minimum Cohen’s *kappa* value for all variables exceeded 0.90, while the intra-class coefficients (ICC) ranged from 0.96 to 1 and standardized typical errors varied from 0.02 to 0.11, demonstrating high inter-rater reliability [27, 28].

### Procedures and statistical analysis

Match statistics from the players and their 52-week world ranking in each slam were initially collected into spreadsheets for the calculation of relative quality. Afterwards, the raw data of each considered match performance variable was calculated into either percentage or ratio scale (shown in Fig 1) to avoid the misinterpretation caused by different units and the respective missing values were cleared. A one-way analysis of variance (ANOVA) was used to calculate the differences of all variables among four Grand Slams. Pairwise differences were assessed with Bonferroni *post hoc* test and partial eta squared (η^2^) was used as the effect size estimate. The alpha level for significance was set at p ≤ 0.05. The data package of IBM SPSS Statistics for Windows (Armonk, NY: IBM Corp.) was used for the ANOVA and the following cluster analysis.

Afterwards, according to the methods of O’Donoghue and Cullinane [24], the relative quality of for each player and their opponents was then calculated using their 52-week world ranking through the following two steps:

1. Using the equation to estimate the round of a slam that a player is expected to reach based on her ranking [29]:
  $$R_{X}=8-{log}_{2}({Rank}_{X})$$
  where *Rank*_X_ is the ranking of player X and *R*_X_ is the round she is expected to reach.
2. Determining the relative quality (RQ) for each match played by player X and player Y:
  $$RQ=R_{X}-R_{Y}$$

Pearson correlation coefficient was calculated to determine the relationships between RQ and each of those 37 variables in four Grand Slams and the magnitudes of the associations was assessed by a spreadsheet developed by Hopkins [30], applying non-clinical magnitude-based inferences with 99% confidence intervals. Strength of the correlation were interpreted as trivial (0.0–0.1), small (0.1–0.3), moderate (0.3–0.5), large (0.5–0.7), very large (0.7–0.9) and nearly perfect (0.9–1.0) [31]. Magnitudes were defined as unclear when the confidence limits for the correlation coefficient overlapped both substantial positive and negative values, while clear correlation were described according to the following scale: 25–75%, possible; 75–95%, likely; 95–99%, very likely; >99%, most likely [31].

As the previous scale of correlation strengths just provides an inferential range, the common filtering of magnitudes would not help to determine which variables should be ruled out while others were not if they belong to the same scale. Therefore, in order to select the variables that had clearly stronger correlations with RQ in different slams for the evaluation of player’s performance and statistically provide an objective cut-value for filtering higher correlation coefficients from lower ones, a two-step cluster analysis with Euclidean as the distance measure and Schwartz's Bayesian criterion was used to classify the magnitudes of correlations. These data were automatically clustered into weaker (r = 0.089 ± 0.092, min to max: -0.117 to 0.221, n = 90 correlations) and stronger correlations (r = 0.347 ± 0.077, min to max: 0.223 to 0.549, n = 58 correlations).

Finally, the typical performance profiles of all players were established by calculating the percentage evaluation score (%ES) as follows [24]:

1. The simple linear regression equations of selected variables and players’ RQ were built (one variable at a time) to determine the expected value of these variables.
2. Calculating the residual value of expected value and observed value, which stand for how much better or worse a player performed than she was expected to do when competing with a rival of certain ranking.
3. Dividing the residual by the standard deviation of the residuals in the data used in the regression model to give a Z-score.
4. Using the standard normal distribution to determine the area of the probability distribution for the residuals for Z-scores less than the one calculated the last step through the “NORMSDIST” function in Microsoft Excel and multiplying this probability by 100% to obtain the evaluation score (%ES). It means the percentage of matches where a performance variable value would be expected to be lower than the observed value considering the RQ of two competing players.
5. Means of %ES of those variables for all players, Top 10 ranked players and a determined individual were calculated and plotted into radar charts in the Excel.
6. When variables like break points per return game, aces of all serves (%), double faults of second serves (%), etc., had 0 denominator for some player observations, the values for such % performance measure were replaced directly with “0”. The reason for this process is because “zero” in performance is real player’s performance in tennis match, and is still a performance for such variables.

## Results

### Descriptive statistics of all players

Table 1 presents the serve performance of the match variables in the four Grand Slams for all female players. Players won more first and second service points at an average of 66.78% (11.1%) and 46.44% (12.7%) respectively in Wimbledon (W), and served more aces with a mean of 4.84% (5%) and committed more double faults averaged at 13.40% (8.3%) in Australian Open (AO). Moreover, more service winners were obtained in slams of hard courts (AO and US Open). When considering the different serve directions in different serves, players won more points in all slams when the first serves landed in the T zone of deuce court and wide zone of advantage court. But for second serves, higher percentage of wins was achieved when balls were served to the body zone in both of the deuce and advantage court.

**Table 1:** Serve performance of female players in four Grand Slams.

Variable		Australian Open	Roland Garros	Wimbledon	US Open	F	p	partial eta squared
		(n range: 764–998)	(n range: 350–498)	(n range: 392–498)	(n range: 526–744)			
		M (SD)	M (SD)	M (SD)	M (SD)			
Aces of all serves (%)		**4.84 (5.0) #**	**3.37 (3.6) ¶†**	**4.79 (5.3)**	**4.43 (4.7)**	**8.42**	**<0.001**	**0.012**
Service winners of all serves (%)		1.05 (1.6) #∫	0.35 (0.8) ¶†	0.82 (1.3) §	1.19 (1.8)	34.57	<0.001	0.042
First Serve								
First serve in (%)		61.32 (8.4) #∫	63.72 (8.4) †	62.76 (7.9) §	60.42 (8.4)	18.94	<0.001	0.020
First serve points won (%)		64.67 (11.5) #∫	61.92 (11.4) ¶†	66.78 (11.1) §	64.40 (11.8)	14.98	<0.001	0.016
First serve points won	in deuce court (%)	64.70 (14.0) ∫	62.42 (13.5) ¶†	67.71 (13.4)	65.65 (13.9)	9.62	<0.001	0.014
	in advantage court (%)	64.61 (14.3) #	61.91 (14.2) ¶	66.27 (14.2)	63.98 (14.2)	6.01	<0.001	0.009
	in Wide zone of the deuce court (%)	65.85 (20.6)	62.62 (23.9) ¶†	67.87 (17.8)	68.04 (23.3)	5.39	0.001	0.008
	in T zone of the deuce court (%)	67.55 (21.3)	63.93 (27.7) ¶†	69.68 (20.5)	68.80 (24.7)	4.41	0.004	0.006
	in Body zone of the deuce court (%)	52.60 (30.5) #	58.78 (21.6) ¶	49.68 (36.9) §	58.02 (24.2)	9.81	<0.001	0.014
	in Wide zone of the advantage court (%)	66.65 (24.6)	63.48 (24.5)	67.95 (21.6)	65.28 (26.1)	2.40	0.066	0.004
	in T zone of the advantage court (%)	64.35 (22.0) #	59.15 (26.9) ¶†	64.86 (20.8)	64.97 (24.8)	5.43	0.001	0.008
	in Body zone of the advantage court (%)	49.56 (33.6) #‡	57.78 (24.5) ¶	46.89 (38.0) §	56.92 (26.7)	13.11	<0.001	0.019
**Second Serve**								
Second serve points won (%)		45.09 (11.9)	45.54 (12.4)	46.44 (12.7)	45.40 (12.5)	1.36	0.252	0.001
Double Faults of second serves (%)		13.40 (8.3) #	11.68 (8.7) †	12.40 (8.5)	13.30 (8.3)	4.25	0.005	0.006
Second serve points won	in deuce court (%)	52.40 (17.6)	50.10 (17.5)	52.13 (18.03)	52.56 (17.8)	1.66	0.173	0.002
	in advantage court (%)	52.20 (19.2)	52.84 (19.0)	54.29 (18.7)	51.81 (17.8)	1.52	0.207	0.002
	in Wide zone of the deuce court (%)	40.86 (40.0)	37.59 (39.8)	44.55 (36.3)	39.11 (41.8)	2.21	0.085	0.003
	in T zone of the deuce court (%)	44.06 (34.8) #	36.67 (37.7) ¶	44.13 (34.7)	40.15 (39.0)	4.19	0.006	0.006
	in Body zone of the deuce court (%)	50.18 (27.5)	49.59 (23.3)	50.57 (32.5)	50.25 (23.7)	0.08	0.969	0.000
	in Wide zone of the advantage court (%)	44.92 (34.8)	49.30 (35.3) †	49.11 (31.8) §	40.71 (37.4)	6.10	<0.001	0.009
	in T zone of the advantage court (%)	41.79 (39.8) #‡	31.79 (40.0) ¶	41.89 (38.6)	35.40 (42.3)	6.90	<0.001	0.01
	in Body zone of the advantage court (%)	47.94 (29.5)	50.48 (27.7)	47.07 (35.8)	48.96 (24.2)	0.98	0.402	0.001

Note: the following legends denote significant difference between paired Grand Slams

# Australian Open (AO) vs. Roland Garros (RG)

∫ AO vs. Wimbledon (W)

‡ AO vs. US Open (US)

¶ RG vs. W

† RG vs. US

§ W vs. US.

Table 2 depicts the performance of return, net point, breakpoint, efficiency and distance covered. More points were won when returning first serves at an average of 38.08% (11.4%) in Roland Garros (RG), and AO was the slam where players had higher percentage of return winners, as well as the return unforced errors, with a mean of 5.19% (5.5%) and 8.69% (7.7%) respectively. The performance of returning the second serves is similar in four events.

**Table 2:** Return, net, break point, efficiency and physical performance of female players in four Grand Slams.

Variable	Australian Open	Roland Garros	Wimbledon	US Open	F	p	partial eta squared
	(n range: 520–998)	(n range: 60–498)	(n range: 272–498)	(n range: 284–744)			
	M (SD)	M (SD)	M (SD)	M (SD)			
**Return Performance**							
Return points won of total returns (%)	43.03 (9.8) ∫	44.13 (9.6) ¶	40.91 (9.8) §	43.27 (10.1)	9.81	<0.001	0.011
Returning first serves won (%)	35.33 (11.5) #∫	38.08 (11.4) ¶†	33.22 (11.1) §	35.60 (11.8)	14.98	<0.001	0.016
Returning second serves won (%)	54.93 (11.9)	54.56 (12.1)	53.56 (12.7)	54.60 (12.5)	1.40	0.241	0.002
Return winners of total returns (%)	5.19 (5.5) #∫‡	2.01 (2.8) †	2.51 (2.42)	2.69 (2.7)	95.93	<0.001	0.107
Return unforced errors of total returns (%)	8.69 (7.7) #∫‡	3.28 (3.6)	3.41 (2.9)	4.07 (3.3)	171.23	<0.001	0.177
**Net Performance**							
Net points won (%)	66.58 (20.1)	64.40 (19.2)	64.60 (17.1)	65.59 (18.6)	1.88	0.131	0.002
Net points won in total points won (%)	10.86 (6.7) ∫‡	11.19 (6.3) ¶†	13.5 (7.9)	12.78 (8.1)	18.33	<0.001	0.022
**Break points Performance**							
Break points per return game	0.77 (0.4) ∫	0.79 (0.4) ¶	0.69 (0.4)	0.74 (0.4)	4.42	0.004	0.006
Break points won (%)	45.73 (23.3)	47.71 (23.8)	44.29 (24.4)	46.17 (24.5)	1.76	0.153	0.002
Break points saved (%)	54.05 (23.1)	51.95 (23.8)	55.54 (24.2)	53.83 (24.5)	1.94	0.121	0.002
**Efficiency Performance**							
Winner per unforced error ratio	0.86 (0.6) #∫	1.01 (0.6) ¶†	1.18 (0.8) §	0.90 (0.6)	32.04	<0.001	0.036
Dominance ratio	1.09 (0.5)	1.08 (0.5)	1.14 (1.0)	1.09 (0.5)	1.03	0.38	0.001
**Physical Performance**							
Mean meters run per match (m)	1338.71 (571.7)	1452.19 (600.2)	1289.28 (567.9) §	1423.18 (589.1)	3.21	0.022	0.008
Mean meters run per set (m)	583.76 (198.1)	618.65 (221.0)	558.31 (188.2) §	608.84 (196.8)	3.65	0.012	0.010
Mean meters run per point (m)	9.71 (2.7) ∫	10.16 (2.6) ¶	9.14 (2.4) §	10.18 (2.9)	7.53	<0.001	0.020

The following legends denote significant difference between paired Grand Slams

# Australian Open (AO) vs. Roland Garros (RG)

∫ AO vs. Wimbledon (W)

‡ AO vs. US Open (US)

¶ RG vs. W

† RG vs. US

§ W vs. US.

Similar net performance was shown in all slams although players tended to approach the net more often in Wimbledon with 13.5% (7.9%) of the total points won being achieved in the net. In RG, players won slightly more break points at an average of 47.71% (23.8%) and in W, 55.56% (24.3%) of the break points was saved, higher than the rest of slams. Moreover, it is shown that players owned a higher average dominance ratio of 1.14 (1.0) in Wimbledon, achieving more winners against each unforced error at a ratio of 1.18 (0.8). Players ran more meters in RG with total distance covered, distance per set and distance per points being higher the other slams.

### Results of correlation analysis and cluster analysis

**Fig 2:**
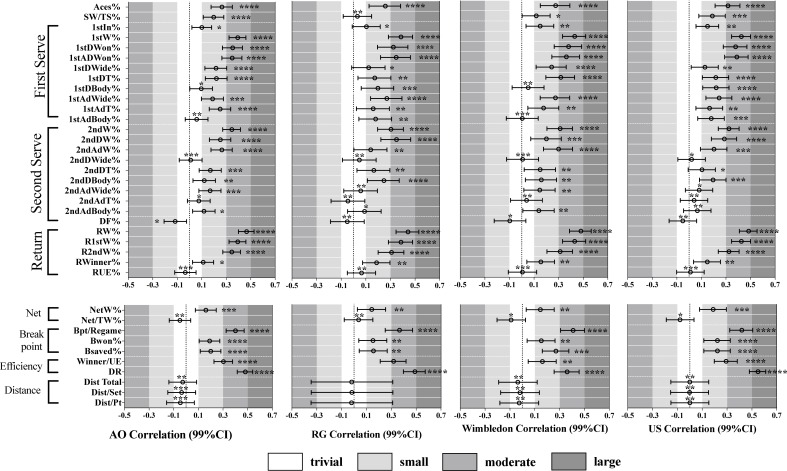
Relationships between match variables and the relative quality (RQ) for female players in four Grand Slams. Asterisks represent the likelihood for the magnitude of true correlation as follow: *possible, **likely, ***very likely, ****most likely. Asterisks located in the trivial zone denote for trivial correlation. Legends: Ace% = Aces of all serves%; SW/TW% = Service winners of all serves%; 1stIn% = First serve in%; 1stW% = First serve points won%; 1stDW% and 1stAdW% = First serve points won in deuce and advantage courts%; 1stDWide%, 1stDT% and1stDBody% = First serve points won in Wide, T and Body locations of the deuce court; 1stAdWide%, 1stAdT% and 1stAdBody% = First serve points won in Wide, T and Body locations of the advantage court; 2ndW% = second serve points won%; 2ndDW% and 2ndAdW% = Second serve points won in deuce and advantage courts%; 2ndDWide%, 2ndDT% and 2ndDBody% = second serve points won in Wide, T and Body locations of the deuce court; 2ndAdWide%, 2ndAdT% and 2nddBody% = second serve points won in Wide, T and Body locations of the advantage court; DF% = double faults of the second serves%; RW% = Total Return points won%; R1stW% and R2ndW% = Return first and second serves won%; Rwinner% and RUE% = Return winner and unforced error of total returns%; NetW% = Net points won%; NetW/TW% = New won of total points won%; Bpt/Regame = Break points per return game ratio; Bwon% = Break points won%; Bsaved% = Break points saved%; Winner/UE = Winner per unforced error ratio; DR = Dominance ratio; Dist. Total = Mean meters run per match; Dist/Set = Mean meters run per set; Dist/Pt = Mean meters run per point. Note: Abbreviations can be applied to the whole text.

The strength of the relationships between match performance variables and relative quality (RQ) in each Grand Slam are presented in Fig 2 by the correlation magnitude, its 99% confidence interval and the likelihood for the magnitude of the true effect. Of all 37 variables, the majority of the them showed clear positive or negative relationship with RQ in all Grand Slams (28 variables in AO, 26 in RG, 27 in W and US). Additionally, within the four slams, some variables differed in their correlation magnitudes with RQ.

Starting from serve performance, aces of all serves showed a small to moderate relationships with RQ in all the slams. And for the first serve performance, the first serve won (%), first serve won in deuce (%) and advantage court (%) had a moderate correlation with RQ in all events. Only in Wimbledon, there is a moderate correlation between first serve points won in T of the deuce court (%) and RQ. Among all variables of second serve performance, the second serve won (%) is the only one that had a moderate association with RQ in four slams while a moderate relation was only shown in RG between second serve won in deuce court (%) and RQ. Only double faults of the second serves (%) in AO was negatively associated with RQ with a possible small magnitude.

Considering the return performance, the moderate to large positive association between the total return points won (%) and RQ in all slams was stronger than that of the returning first and second serve points returns (%).

The net points won (%) performance in all slams were positively correlated with the RQ with a small magnitude while only trivial connection was found between the net points won in total points won (%) and RQ.

Break points opportunity in return game (%) demonstrated a moderate positive correlation with RQ in all Grand Slams. A small positive relation was shown between break points won (%), break points saved (%) and RQ. The winning efficiency was also positively associated with RQ in that the dominance ratio in AO, RG and US had a moderate to large positive correlation with it, and in Wimbledon they were moderately associated; and winner per unforced error ratio showed a small association with RQ in all slams. The total distance covered, distance covered per set and per point had only trivial correlations with RQ with the ones of RG being unclear.

The total of 148 correlation magnitudes (37 in each of four slams) were classified into two groups by the two-step cluster analysis. In the group of stronger correlations, there are 14 variables respectively from AO and RG that belong to this group, and 15 variables respectively from W and US. Therefore, variables with stronger correlations with RQ in each slam were treated separately to calculate the percentage evaluation score (%ES). Table 3 presents the descriptive analysis of these variables and the relative qualities for Top 10 ranked players and Serena Williams in four slams. Meantime, those variables were combined together with those from all players shown in Tables 1 and 2, to establish the typical performance profiles of players.

**Table 3:** Descriptive statistics of match performance and relative quality for Top-10 ranked female players and Serena Williams in four Grand Slams.

Variable	Australian Open		Roland Garros		Wimbledon		US Open	
	Top 10	Serena Williams	Top 10	Serena Williams	Top 10	Serena Williams	Top 10	Serena Williams
	(n = 152)	(n = 25)	(n = 70)	(n = 14)	(n = 75)	(n = 14)	(n = 105)	(n = 18)
	Mean (SD)	Mean (SD)	Mean (SD)	Mean (SD)	Mean (SD)	Mean (SD)	Mean (SD)	Mean (SD)
*Relative Quality (RQ)*	3.3 (2.0)	4.5 (1.8)	3.3 (2.0)	5.0 (1.5)	3.2 (2.1)	4.7 (1.8)	3.2 (2.2)	4.6 (1.5)
Aces of all serves (%)	7.2 (7.1)	16.3 (6.4)	5.2 (4.7)	10.3 (4.5)	7.2 (7.8)	19.4 (7.9)	6.9 (5.9)	14.2 (6.6)
First serve points won (%)	70.3 (11.4)	79.8 (9.8)	68.3 (9.8)	73.2 (7.9)	71.3 (10.7)	81.3 (7.2)	71.5 (10.8)	80.6 (7.1)
First serve points won in deuce court (%)	70.4 (13.6)	79.1 (11.8)	68.7 (11.7)	72.7 (11.0)	72.5 (12.0)	82.1 (10.8)	71.7 (12.7)	82.8 (5.2)
First serve points won in advantage court (%)	70.2 (13.2)	79.9 (11.7)	68.4 (11.9)	73.7 (9.2)	71.8 (13.4)	81.9 (10.1)	71.1 (13.0)	78.1 (12.4)
First serve points won in Wide zone of the deuce court (%)					72.4 (15.9)	78.9 (14.9)		
First serve points won in T zone of the deuce court (%)					75.2 (17.6)	85.3 (14.3)		
First serve points won in Wide zone of the advantage court (%)			72.9 (19.9)	81.7 (10.8)	76.8 (16.6)	92.7 (9.9)	71.9 (25.4)	77.7 (23.4)
First serve points won in T zone of the advantage court (%)	70.4 (22.4)	79.0 (15.5)						
Second serve points won (%)	48.8 (11.5)	51.7 (11.0)	48.6 (11.1)	50.5 (7.4)	50.7 (14.0)	51.9 (11.2)	49.6 (12.4)	52.9 (12.0)
Second serve points won in deuce court (%)	56.2 (17.5)	56.5 (15.4)	56.9 (17.8)	61.1 (16.5)			55.8 (16.1)	63.0 (13.6)
Second serve points won in advantage court (%)	54.7 (19.7)	60.6 (16.4)			62.6 (19.9)	65.4 (13.3)		
Second serve points won in Body zone of the deuce court (%)			56.4 (21.2)	61.6 (19.5)				
Return points won of total returns (%)	48.6 (8.7)	48.7 (7.2)	48.3 (8.6)	49.4 (9.0)	46.5 (8.6)	49.5 (7.1)	48.6 (10.0)	47.6 (7.4)
Returning first serves won (%)	40.2 (10.8)	40.43 (9.5)	41.6 (11.0)	42.8 (9.1)	39.7 (10.6)	41.0 (9.4)	41.2 (13.0)	39.0 (11.0)
Returning second serves won (%)	61.0 (11.4)	60.06 (11.3)	58.9 (9.3)	60.9 (11.0)	57.0 (11.5)	62.2 (11.1)	59.3 (10.3)	59.6 (6.7)
Winner per unforced error ratio	1.1 (0.5)	1.38 (0.6)	1.2 (0.7)	1.5 (1.1)			1.2 (0.6)	1.5 (0.6)
Dominance Ratio	1.4 (0.8)	1.87 (1.6)	1.3 (0.5)	1.5 (0.8)	1.6 (2.1)	1.9 (1.2)	1.4 (0.6)	1.7 (0.6)
Break points per return game	1.0 (0.4)	1.0 (0.3)	0.9 (0.4)	0.9 (0.3)	0.9 (0.4)	0.9 (0.3)	0.9 (0.4)	1.0 (0.4)
Break points won (%)							51.6 (19.7)	47.7 (19.7)
Break points saved (%)					58.1 (30.0)	74.3 (31.0)	59.0 (28.5)	68.0 (33.7)

Blank cells denote the variables that did not had stronger correlations with RQ in determined slams according to the cluster analysis and were not considered in those slams to evaluate players’ performance.

### Profiles of typical performance for player

**Fig 3:**
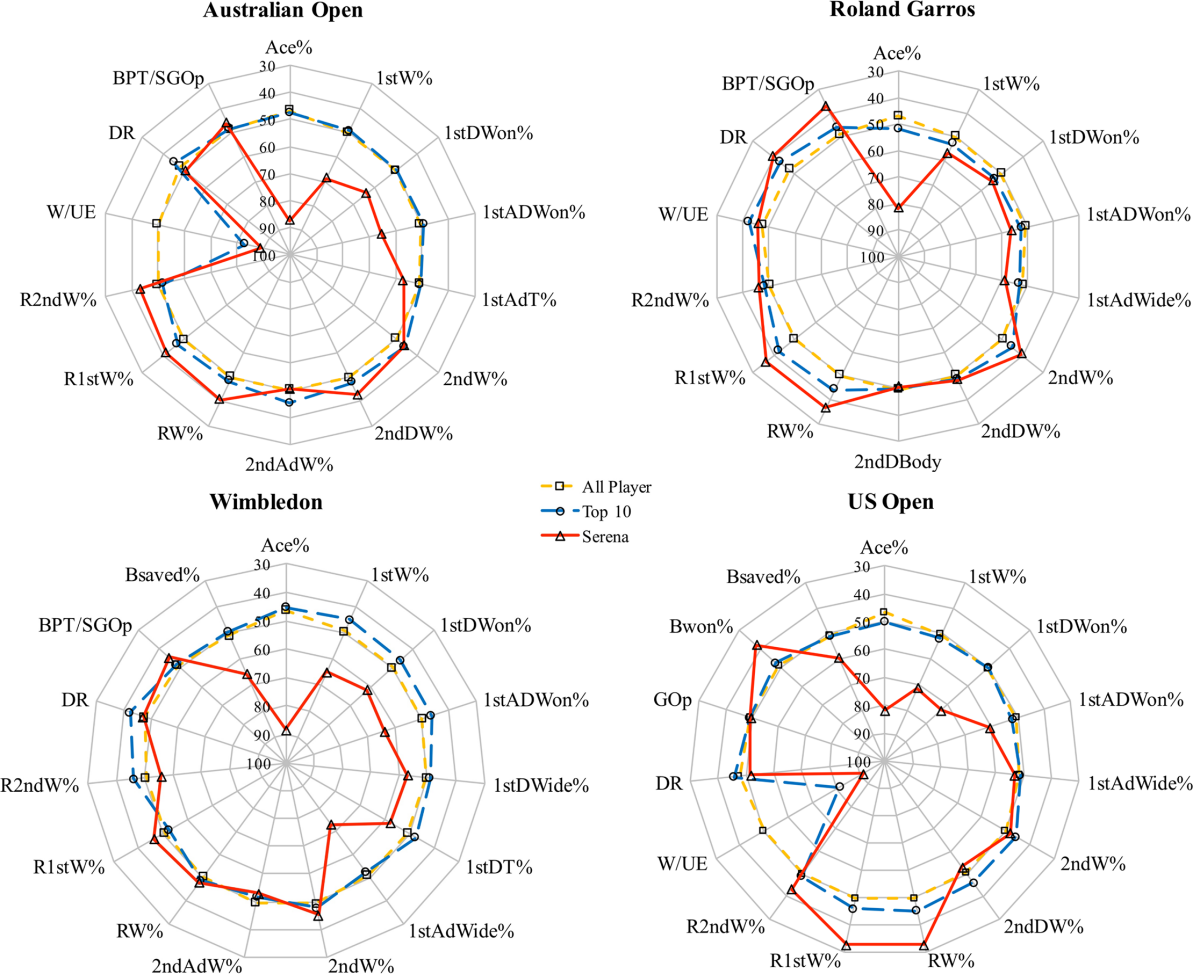
Performance profiles of percentage of evaluation score (%ES) for: All female players, Top-10 players and Serena Williams in four Grand Slams.

Fig 3 presents the means of the %ES scores for all variables (AO: 14, RG: 14, W: 15, US: 15) that were selected. The %ES of all players averaged round 50%. And when it comes to those of Top-10 players and Serena Williams, a clearly visual inspection detected the performance differences among them. In all slams, Serena Williams showed a dominant performance in serving more aces of total serves, considering of the quality of her opponents, whereas the Top-10 players only had a similar performance to that of all players. Better performance in her efficiency of winner per unforced error ratio was demonstrated in all slams except for Roland Garros, where Serena Williams performed inconsistent or similar like other player. Worse or equal %ES to that of general player was shown in her return and second serve performance. Moreover, results also showed that Serena Williams had more stable performance in saving more break points in fast surfaces events (AO, W and US), but not in slow surface(RG).

## Discussion

This study aimed to assess the female players’ performance in four Grand Slams and build the typical match performance profiles based on relative quality (RQ). Performance variables that were more associated with RQ were identified from all variables. This is the first study to identify the correlation between match performance variables and RQ and to assess the performance of female players in four Grand Slams via the regression based method [22, 24].

Effects of game location were observed across a range of performance variables all Grand Slams that implies the baseline oriented match pattern and the importance of serve and return efficiency in female tennis games. The majority of variables showed a positive correlation with relative quality, but not physical characteristic, which was revealed to be unaffected by quality of opponents. Different magnitudes of correlations for each variable in distinct Grand Slam suggested the joint effect of game location and quality of opposition. The analyses of typical performance underlined the usefulness of applying percentage evaluation scores to interpret player’s performance.

The results of the current study confirm that match location had an influence on the performance of female players and their strategies in Grand Slams. Previous research showed that the difference of court surfaces, court dimension, court speed, ball types, temperature and humidity might cause players’ distinct adaptation to the courts, thus resulting in their different performance[1, 8, 13, 25, 32–34]. Nonetheless, more factors such as surface abrasion, venue altitude and the distance between court and audience should be further looked into to determine and quantify the effects.

### Serve and return performance

The importance of serve and return performance in four Grand Slams have been widely addressed in previous studies [7, 10, 12, 14, 35] and been treated as the two most important performance variables in tennis match [13, 25, 36]. Consistent with former studies [13, 25], the results indicated that in all Grand Slams, female players had an optimal first serve in percentage of round 60% and still used a “fast-weak” serving strategies. This possibly due to that they were more aggressive in the first serve by hitting more flat and faster balls, and conservative in the second by using slower and more topspin serves [13, 37]. Accordingly, receivers won less points when returning the first serves but more points when returning the second serves in all slams, which showed that they were more prepared to return aggressively the second serves and take control of the points [25]. However, it should be highlighted that female players had higher percentage of winning first serve returns in Roland Garros than the other slams probably because of the slower surface speed that permits returner to have more time to react [13, 38]. This finding can also be due to the fact that players achieved less aces and serve winner in Roland Garros. Therefore, the strategy of the fast first serve would not be as effective as in other slams. Although slow surface also determined that returning players in RG could not hit more return winners neither, as shown in the results, they managed to reduce the return unforced errors and counteract the dominance of the servers over their first serves [14].

Nevertheless, results demonstrate that in Australian Open and US Open, players committed more double faults and return unforced errors while having more service and return winners than other slams. On the one hand, this would indicate firstly that in these two slams, female players had a preference of “strong-strong” serve strategy that although enable them to be more aggressive at serves and win more points, but also made them to be too aggressive to make more double faults; and on the other hand, that they carefully prepared their positions in return with good anticipation, especially in Australian Open to win attain more return winners, which corroborates the finding of O’Donoghue and Brown (2008). Whilst in terms of similarly high percentage of return unforced errors, two possible explanations would be: (i). Serve returners tried to dictate the point at the onset of the second serve to put serving opponents under pressure so that they made more mistakes in returning; (ii). Players’ concentration and reaction in return was impaired by the high temperature and humidity in the period of celebrating Australian and US Open because the two slams are held in hot environmental conditions [15, 39] and the effect of physical fatigue, hypo-hydration, hyperthermia (increased body temperature), unpredicted match length with repeated movements and psychological stress players perceive would jointly impair their performance [15, 34, 40, 41]. Further research could investigate the relationship between hitting a return winner or unforced error and player’s return position, physiological and psychological responses, serve speed and angel of the serve [10].

Additionally, results of the spatial distribution of the serves showed that in professional female matches, players generally won more points by placing the first serves to the T and wide zones of service box, independent of the deuce or advantage side. In that way, they could not only increase their possibility of serving aces [10], but also made the receivers feel more difficult to return [4]. However, trying first serves into the body zones might not seem to be a bad option in female matches [4], especially in Roland Garros and US Open where players could still won around 58% of total serves points in body zones. Concerning the second serves, the finding of the study agrees with Hizan et al. (2015) in that female players favored serving to body zone of the receivers and won more points. Because this strategy could avoid the receiver to use their double-handed backhand to attack the serve. But the results also showed that placing second serves to the backhand of the opponents was also as effective as serving to forehand side (T zone).

### Technical and physical performance

Results has demonstrated that female players had merely around 10% of total points won that were won in the net, which suggests that professional female players still remained conservative in approaching the net and preferred baseline strategy in all Grand Slams to compete [8] although they had good effectiveness in net performance, winning 65% of all net points. In terms of break point performance, female players obtained similar break opportunity per game, break points conversion and break point saved percentage to the ones of male players [6, 11]. When Players had better efficiency in Wimbledon, they may win more points rather than making unforce errors and had higher dominance ratio of achieving more points in return game rather than losing points in own service game. A potential explanation is that in Wimbledon, the faster surface speed shortened the rally number and increased the rally rate, as a result it was easier for players to strike more direct winners [8, 12].

Similar to the finding of Reid et al. (2016), female players covered approximately 1289m to 1452m per match, 558m to 618m per set and 9.14m to 9.71m per point, with Wimbledon being the one where players ran less meters and Roland Garros being the one players ran more distance. But the difference of distance covered among all slams was not that obvious because it was more dependent on the game and score-line of the match [9]. Limited formers studies have already considered the movement patterns of male professional players in real match situations and inter-player relationship [18, 26, 42], but few has been done with female players [9]. Therefore, it is necessary that future researches focus on the movement characteristics of female players based on different match situations and opponent’s qualities.

### Relationship between match variables and relative quality and cluster analysis

The findings of the current study reinforced the O’Donoghue’s (2009) theory and provided more information in that the difference between players’ strength (ranking) also influenced female player’s performance on many aspects.

First of all, results were in agreement with O’Donoghue and Cullinane (2011) on that the first serve in percentage is not correlated with relative quality (RQ) in any of the four slams; and percentage of serve and return points won are positively associated with RQ in all events. Meanwhile, other serve and return performance variables were also encountered to have positive correlation with RQ in all slams, such as percentage of first serve points won in deuce and advantage courts, percentage of second serve points won in deuce and advantage courts, percentage of aces and return winners. However, the percentage of return unforced error and the percentage of second serve points won in Wide zone of the deuce court had trivial correlation with RQ. Moreover, there were variables like percentage of service winners and double faults, which were revealed to have clear association with RQ in certain slams but not in all of them.

Secondly, in terms of the efficiency and physical variables, there were no clear relationships between the percentage of net points won in total points won, the distance covered in match, set and point and RQ in all Grand Slams. While net point won (%), break point per return game, break point won (%), break point saved (%), winner per unforced error ratio and dominance ratio were all positively associated with RQ.

The quality of opposition is one of the contextual variables that have been widely considered in team sports to evaluate and explain the performance of interested teams against different level of opponents [33, 43–45] but there is a paucity of investigation in tennis over this topic [19, 24, 46]. Gosseens et al. (2015) grouped the tennis players into 7 categories according to their world ranking and tried to predict the winning probability of male and female players in Grand Slams; Reid et al. (2010) used the match statistics of male tennis player tournament from Association of Tennis (ATP) to decide what performance variables best indicates of player’s top-100 ranking. However, only O'Donoghue [47] considered in the interacting performance theory that the strength and the type of opponents had major effects on the performance outcome of tennis players and O'Donoghue and Cullinane (24) found the meaningful correlations between match statistics and relative quality in Grand Slams male games, which gave more evidence on how quality of opposition could vary the performance of players.

The results of correlation not only suggested that ranking of professional female players could be used to evaluate the player performance [48] and the comparative strength between two players is a good predictor of their match behaviors [24]; but also indicated the influence of different court surfaces on how players planned their strategies against players of various levels [8].

The results of two-step cluster analysis supported the last statement as well in that not all variables in the stronger correlation group were the same ones in every slam, and some of their strengths of correlation varied from one to another. For example, the percentage of second serve points won in Body zone of the deuce court was the variable that had stronger relationship in Roland Garros as it is a preferred zone for female players to place their second serves [4]. Also, it could be implied that in further research, the selection of performance variables should could be altered by the necessity of coach and players, according to different match and opponents [22].

### Performance profiles using percentage evaluation score (%ES)

In the final part of the current study, we built the typical performance profiles for Top-10 ranked players and Serena Williams, using the %ES of variables from stronger correlation group. Former profiling techniques [23] was questioned for not considering the opposition effects when establishing performance profiles and individual performance may sometimes be underestimated [22]. Therefore, the study applied the approach of O’Donoghue and Cullinane (2011) to describe the players’ performance in relation to their strength. The approach is a more suitable profiling technique in that it gives an evaluation score to the player’s performance based on her difference in ranking with opponents. Another merit of the approach is that: with only one unit of measurement percentage, the %ES of variables that are originally different in unit, could be readily visualized in coaching process. This avoids the normalization of performance variables, which sometimes omits the individual performance. Besides, there are certain variables the coach can easily understand without being normalized in the practical scene [21, 22]. Therefore, from a practical perspective, the study suggests that visualized %ES can be provided along with actual match statistics in coaching process to have better understanding of player’s performance.

While the study has advanced the understanding about female players’ performance in different Grand Slams and provided more information on the typical performance of players using ranking-based approach, there are some limitations that should be addressed. Firstly, the match statistics of just two seasons from Roland Garros and Wimbledon were used and this would lead to a possible results bias when comparing the performance among different Grand Slams. Besides, although the study covered a more comprehensive range of match performance, it failed to account for players’ performance in tiebreaks, which provides substantial information on how they behaved under critical moments. Additionally, some players were repeatedly used as independent sample due to the that their performances were conditioned by various contextual factors [33], but sampling the same players more than once across different Grand Slams is a possible limitation. Moreover, considering only world ranking is insufficient to account for the quality of opposition, and it is preferred that future study could look into others player characteristics such as anthropometric features, handedness, backhand style, experience, etc. [20]. In relation to the last point, as the current study failed to take into account the influence of handedness on players’ serving strategy, so that caution should be taken when generalizing the findings to left-handed players. Lastly, rather than considering the absolute performance values, it is suggested that the relationships between differences in performance of two competing players and their relative quality should be explored in the future study.

## Conclusion

The current study provides preliminary assessment on the match-play performance of female tennis players by: (i) analyzing the performance of professional female tennis players in four Grand Slams using match statistics; (ii) measuring the relationships between performance variables and relative quality; (iii) building the typical match performance profiles of tennis players during Grand Slams with the consideration of opposition effects. Results provide noble insights into the performance of professional tennis players and could improve the coaching process. Generally, female game in all Grand Slams remained to be a contest over baseline, although players were shown to have good efficiency at net. Relative quality was found to have an impact on various match performance variables related to serve and return, break point, net point and efficiency except for distance covered. These findings evidenced the joint effect of courts and opponents on players’ match behaviors. And finally, using the percentage evaluation score (%ES) to establish typical performance profiles for players would facilitate a more reasonable assessment of match performance and help to set practical training and match targets. The profiles of professional players could serve as a benchmark for juniors who aspire to achieve a professional success. Moreover, in light of player’s long-term development, it would be optimal if coaches and performance analysts could use this profiling technique to keep a longitudinal monitoring of their players’ technical, tactical and physical performance, exploring important information about individual variation within different match contexts.

## Supporting information

S1 File Supplementary table for data sharing (female player statistics in Grand Slams). (XLSX)
